# Incongruent reduction of dopamine transporter availability in different subgroups of alcohol dependence

**DOI:** 10.1097/MD.0000000000004048

**Published:** 2016-08-19

**Authors:** Che-Hung Yen, Mei-Chen Shih, Cheng-Yi Cheng, Kuo-Hsing Ma, Ru-Band Lu, San-Yuan Huang

**Affiliations:** a Graduate Institute of Medical Sciences, National Defense Medical Center; b Department of Neurology, Tri-Service General Hospital, National Defense Medical Center; c Department of Psychiatry, Tri-Service General Hospital, National Defense Medical Center; d Department of Nuclear Medicine, Tri-Service General Hospital, National Defense Medical Center; e Department of Anatomy and Biology, National Defense Medical Center, Taipei; f Institute of Behavior Medicine, College of Medicine, National Cheng Kung University, Tainan, Taiwan, ROC.

**Keywords:** alcohol dependence, cognitive function, dopamine transporter, major depression

## Abstract

The dopamine transporter (DAT) plays a crucial role in the pathogenesis of alcohol dependence (AD) and major depression (MD), and males have more risk factors for the development of AD. However, imaging studies on brain DAT availability in males with AD comorbid with MD (AD/MD) are limited, and the association of DAT availability with cognitive function and depressive scores in patients with AD/MD has not been analyzed. Hence, this study examined the relationship between brain DAT availability, cognitive function, and depressive symptoms in different subgroups of males with AD.

Single-photon emission computed tomography imaging with ^99m^Tc-TRODAT-1 as a ligand was used to measure striatal DAT availability in 49 patients with AD (28 pure AD and 21 AD/MD) and 24 age- and sex-matched healthy volunteers. The Wisconsin Card Sorting Test (WCST) and 17-item Hamilton Depression Rating Scale were used to assess neurocognitive function and depressive scores, respectively. Patients with AD showed a significant reduction of DAT availability in 3 brain regions (*P* < 0.001), and this reduction was more pronounced in the patients with pure AD compared to healthy controls. The patients with AD showed significantly poorer performance on the WCST, but only in the control group was DAT availability significantly negatively correlated with total errors and perseverative errors (*P* < 0.001).

These preliminary findings suggest that DAT availability is associated with neurocognitive function, and incongruent reduction of DAT may play a pathophysiological role in different subgroups of AD. In addition, decreased DAT availability may be associated with the severity of depressive symptoms in patients with AD/MD.

## 1 Introduction

There is much evidence to suggest that dysfunction of the central dopaminergic system is a crucial factor in the development of alcoholism^[1,2]^ and depressive disorder.^[3]^ Low concentrations of homovanillic acid (a metabolite of dopamine) have been found in the cerebrospinal fluid of individuals with early-onset alcoholism^[4]^ and depressive patients.^[5]^ Hence, reduced dopaminergic transmission may contribute to specific behavioral symptoms of addiction and depression. The dopamine transporter (DAT) is a membrane-spanning protein^[6]^ responsible for the reuptake of dopamine from the synaptic space into the presynaptic neuron. Striatal dopamine levels, which reflect the intraneuronal vesicular storage pool of dopamine, were reduced in DAT knockout mice.^[7]^ Therefore, the DAT plays a crucial role in the regulation of synaptic dopamine levels.

Alcohol is a highly addictive substance, and continuing alcohol abuse disrupts the dopaminergic system in the brain. Previous brain imaging studies using single-photon emission computed tomography (SPECT) found a reduction in striatal DAT levels in patients with alcohol dependence (AD).^[8–10]^ In addition, studies using postmortem human whole-hemisphere autoradiography also suggest that DAT density is lower in alcoholics.^[11]^ Depression is a common comorbidity in patients with AD.^[12]^ Dopaminergic abnormalities within the limbic system were also observed in several animal models of depression,^[13,14]^ and drugs that reduce dopamine levels may lead to depressed mood.^[15]^ Based on evidence from previous studies, dopaminergic dysfunction may be one of the common mechanisms of pathogenesis in both AD and MD.

Reduced dopamine metabolism in the frontal areas, including the anterior cingulate gyrus and orbitofrontal cortex, has been found in patients with AD^[4]^; these regions belong to areas of the cortex associated with executive function and attention. In previous studies, cognitive deficits in attention, cognition, visuospatial skills, and memory have been recorded in patients with AD.^[16]^ The Wisconsin Card Sorting Test (WCST) is a well-established test for measurement of impaired cognitive flexibility.^[17]^ However, investigation of differences in striatal DAT availability as it relates to the WCST within different subgroups of AD has been limited.

MD is a common comorbidity of AD and dopaminergic dysfunction plays a critical role in the development of AD and MD. Hence, it is important to elucidate the role of the DAT in AD with and without MD. The prevalence rates for alcohol use disorders differ widely between males and females, and males are more likely to possess the exogenous risks and endogenous vulnerabilities associated with alcohol abuse.^[18]^ Therefore, this study aimed to investigate striatal DAT availability and its relationship to cognitive function specifically in male patients with pure AD and AD/MD, as compared to healthy male controls. We hypothesized that the 3 groups would show differences in both striatal DAT availability and the WCST scores, and that striatal DAT availability would correlate with the WCST scores. In addition, we investigated a putative association between the depressive score and striatal DAT availability in the human brain.

## 2 Materials and methods

### 2.1 Participants

Individuals who voluntarily sought treatment for AD at the Tri-Service General Hospital, National Defense Medical Center, Taiwan, between 2010 and 2014 and who met inclusion/exclusion criteria were eligible to participate. All participants were older than 20 years of age and all patients had been diagnosed with either AD, MD, or both based on the Diagnostic and Statistical Manual of Mental Disorders, 4th ed., text revision. In addition, participants were screened for use of and dependency on other illegal substances. Each patient and control were initially assessed by an attending psychiatrist and then interviewed by a well-trained psychologist, using the Chinese version of the Modified Schedule of Affective Disorder and Schizophrenia-Lifetime (SADS-L) to screen out other psychiatric conditions. The severity of depression was measured with the 17-item Hamilton Depression Rating Scale (HDRS); each patient with AD and comorbid MD (AD/MD) had an HDRS score ≥18, but patients with pure AD and controls scored <10 on the HDRS on the day of the brain imaging study. Subjects were drug naïve or had ceased psychotropic medication over 1 month prior to the study, but lorazepam (2–8 mg/d) was permitted in order to prevent alcohol withdrawal before the imaging study. Patients were also excluded if they had a comorbid axis I or II mental disorder (except AD and MD), unstable medical conditions that could alter cerebral function including malignancy, cardiovascular disease, renal and hepatic diseases, head trauma with loss of consciousness, or any other neurological diseases.

The patient group consisted of 49 AD subjects recruited during intoxication or withdrawal who had consumed alcohol during the previous 72 h. With respect to the classifications of AD, our previous studies revealed that pure AD and AD/MD are discrete and genetically well-defined subgroups of AD.^[19,20]^ Therefore, subjects with AD were classified into 2 subgroups including pure AD, which was diagnosed in 28 individuals, and AD/MD, which was diagnosed in 21 individuals.

This study enrolled only male participants in order to avoid the potential confounding effects of sex on striatal DAT availability,^[21,22]^ because males present with more alcohol-associated problems than females.^[18,23]^ The control group consisted of 24 psychiatrically healthy male volunteers. Each subject was interviewed by a well-trained psychiatrist using SADS-L to exclude the possibility of major physical illness, psychiatric diseases, or substance abuse. None of the subjects was taking medications that could affect the central dopaminergic system during the study. The study was approved by the Institutional Review Board for the Protection of Human Subjects at the Tri-Service General Hospital (TSGHIRB No. 099-05-017), a medical teaching hospital belonging to the National Defense Medical Center in Taipei, Taiwan. Each participant provided written informed consent before the research was initiated. The procedures and raw data were monitored by the Intuitional Review Board at TSGH and National Research Program for Biopharmaceuticals. All raw data deposition in the laboratory of TSGH for the protection of human subjects, but these dada permit study members to make data available under the procedures of TSGHIRB.

### 2.2 Imaging acquisition and data analysis

The SPECT procedure with ^99m^Tc-TRODAT-1 for DAT imaging has been described elsewhere.^[10,24]^ TRODAT-1 kits were provided by the Institute of Nuclear Energy Research (Lungtan, Taiwan). All subjects consumed a low-protein diet for 24 h before the ^99m^Tc-TRODAT-1 injection and SPECT measurement. The SPECT images were analyzed along the level of the canthomeatal line. The composite image from the 3 highest-activity basal ganglia slices from a given participant was co-registered with the corresponding computed tomography (CT) image to exclude possible brain lesions and to delineate standardized regions of interest (ROIs) for the caudate, putamen, and striatum. Magnetic resonance imaging was used to recheck the brain lesions if the CT scan produced inconclusive results. The defined ROIs were then manually applied to the other SPECT slices from the respective participant.^[21]^ The occipital cortices, which had low DAT concentrations, were also drawn in the same way and served as background areas. The uptakes of ^99m^Tc-TRODAT-1 in various brain regions were measured 4 h after injection, and the specific uptake ratio (SUR) of each region was calculated using the following equation: (ROI_target_ − ROI_reference_/ROI_reference_). The researcher drawing the ROIs on the images was blinded to the subject group.

### 2.3 Wisconsin Card Sorting Test

A computer-aided WCST was administered by an experienced psychologist on the same day as the SPECT scan. During the WCST, all participants were asked to match response cards with 4 key cards along 1 of 3 perceptual dimensions (color, form, or number). Verbal feedback (right or wrong) was given without revealing information about the dimensions. The test requires subjects to find the correct classification rule by trial and error. Once the subject chooses the correct rule, the subject must keep this sorting principle across changing stimulus conditions. After 10 consecutive correct matches, the classification principle changed without warning, demanding flexibility in set-shifting. There were 128 response cards during the test, and the test proceeded until 6 sorting categories had been acquired or until all the cards had been sorted. In accordance with the WCST manual,^[25]^ the following parameters were analyzed: total corrects, total errors, perseverative errors, nonperseverative errors, categories completed, and failure to maintain set.

### 2.4 Statistical analysis

All statistical analyses were performed using SPSS 19.0 software (SPSS Inc., Chicago, IL). The threshold of significance was defined as a *P* value ≤ 0.05 (2-tailed). Two independent samples with continuous variables were compared using the Mann–Whitney *U* test. The Kruskal–Wallis test was used to analyze differences in age, education, and the number of cigarettes smoked per day among the 3 groups (pure AD, AD/MD, and controls). Smoking status was tested using the Pearson χ^2^ test. As this study was based on multiple comparisons, results could have arisen due to Type I errors. Therefore, Bonferroni corrections were applied to reduce issues related to family-wise error rates. Given that each participant had 3 correlated SURs (caudate, putamen, and striatum)—a multiple linear regression using the generalized estimating equation (GEE) was applied to adjust clustering within individuals. The effects of specific factors on the SURs were assessed with an exchangeable working correlation structure.

Spearman rho was carried out to examine the cross-sectional association between DAT availability and the WCST and HDRS parameters. Correlations between the SURs and other parameters (e.g., age, daily alcohol intake, and duration of AD) were also assessed with Parson correlations.

## 3 RESULTS

### 3.1 Participants’ demographic characteristics and WCST parameters

Twenty-eight patients with pure AD (mean age 43.29 ± 10.32 years), 21 patients with AD/MD (mean age 38.57 ± 6.97 years), and 24 healthy controls (mean age 39.21 ± 8.87 years) were enrolled in this study. These subjects underwent SPECT scanning and completed the WCST and HDRS. Their demographic data are presented in Table 1. There were no significant differences in age among patients with pure AD, AD/MD, or healthy controls (*P* = 0.211). Notably, years of education and the numbers of cigarettes smoked per day were significantly different among the 3 groups (*P* < 0.001). The HDRS score was significantly higher in the AD/MD group than the pure AD group (*P* < 0.001) (Table 1).

**Table 1:**
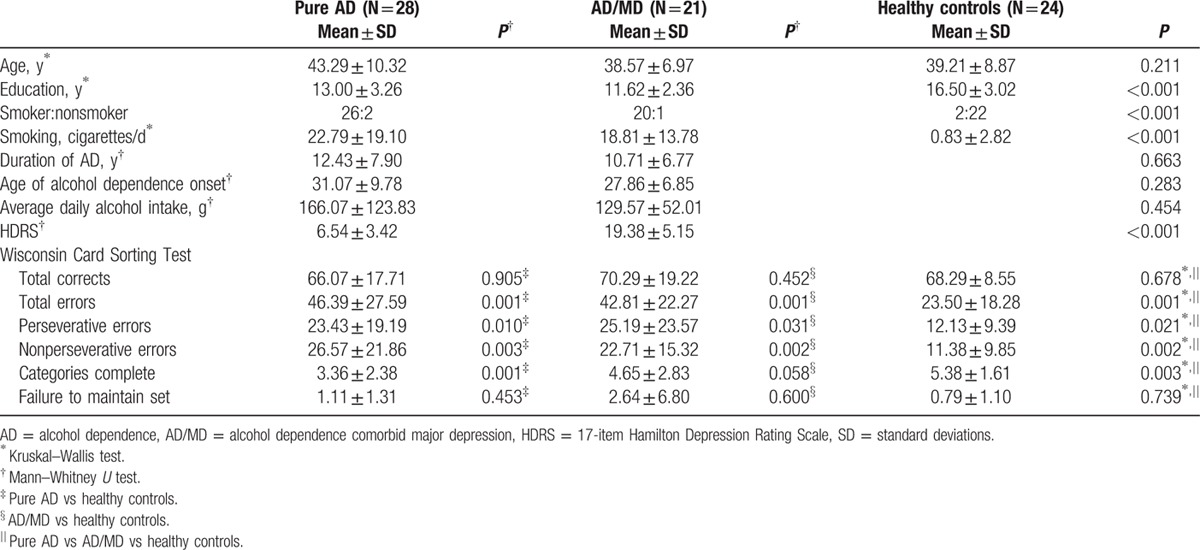
Demographic characteristics and parameters of Wisconsin Card Sorting Test in people with pure AD, AD/MD, and healthy controls.

Patients with AD were significantly impaired on several WCST measures, including total errors (*P* = 0.001 for pure AD, AD/MD, and total AD), nonperseverative errors (*P* = 0.003 for pure AD; *P* = 0.002 in both AD/MD and total AD), and categories completed (*P* = 0.001 for pure AD; *P* = 0.003 for total AD). These results were significant after Bonferroni correction (conservative *P* value would be 0.05/14 = 0.0036). In contrast, there were no significant differences in the WCST measures of total correct, perseverative errors, or failure to maintain set (Table 1).

### 3.2 DAT availability in healthy volunteers and patients with AD

**Figure 1:**
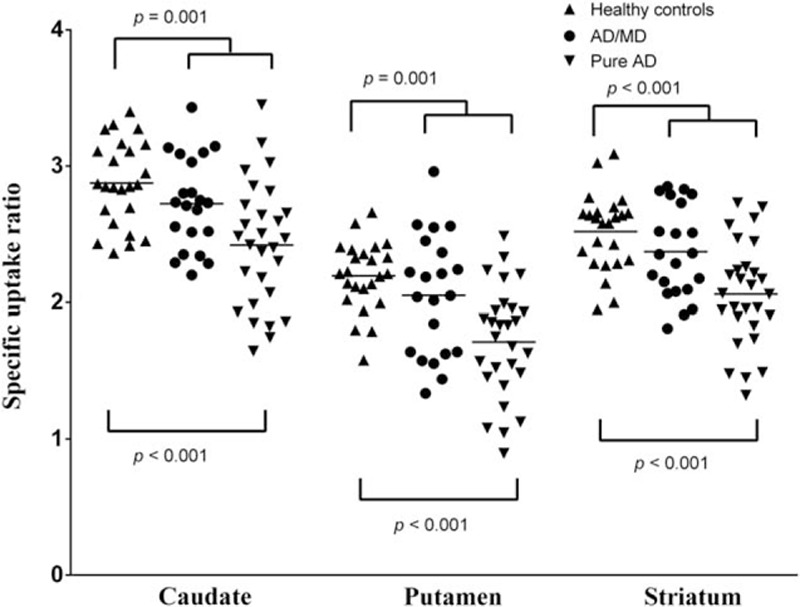
The scatter plot of SUR of DAT in caudate, putamen, and striatum calculated from ^99m^Tc-TRODAT-1 SPECT in healthy control individuals (n = 24), alcohol dependence comorbid major depression (n = 21), and pure alcohol dependent subjects (n = 28). (Conservative *P* value would be 0.05/12 = 0.0042.) Horizontal bars indicate mean value of SUR. DAT = dopamine transporter, SPECT = single-photon emission computed tomography, SUR = specific uptake ratio.

In the healthy volunteers, the mean values of DAT availability in the striatum, putamen, and caudate were 2.52 ± 0.28, 2.19 ± 0.25, and 2.87 ± 0.31, respectively. Patients with total AD showed significantly lower DAT availability than healthy controls in the 3 brain regions (caudate, putamen, and striatum, *P* ≤ 0.001, respectively; Table 2). The values of DAT availability in patients with AD were 2.19 ± 0.39 for striatum, 1.86 ± 0.45 for putamen, and 2.55 ± 0.43 for caudate, respectively. Moreover, this effect was more pronounced in the pure AD group compared with the control group (caudate, putamen, and striatum, *P* < 0.001) (Fig. 1, Table 2).

**Table 2:**
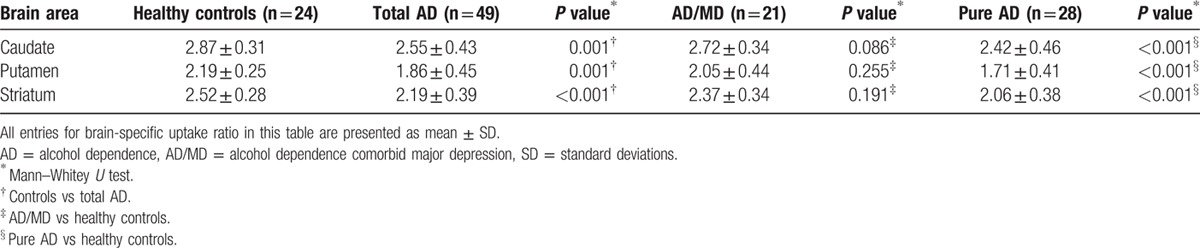
Brain-specific uptake ratio of healthy controls, AD/MD, and pure AD subgroups.

The GEE analysis was used to explore the effects on the SURs of variables such as age, education, smoking status, number of cigarettes smoked per day, and group. The caudate and putamen SURs differed significantly from those of the total striatum (*P* < 0.001). When analyzing the correlation between age and SUR, a significant effect was found (*P* < 0.001). Number of cigarettes smoked per day did not have any significant association with SUR (Table 3).

**Table 3:**
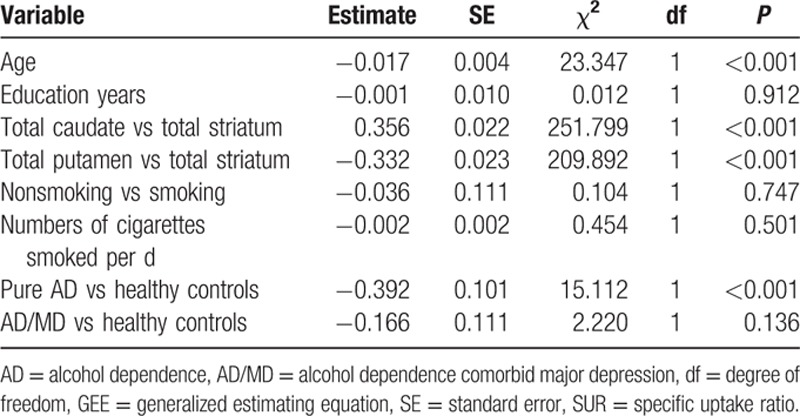
Effects of age, education years, smoking status, brain regions, and groups on the SUR using the GEE method.

### 3.3 Relationship between WCST parameters and DAT availability

**Figure 2:**
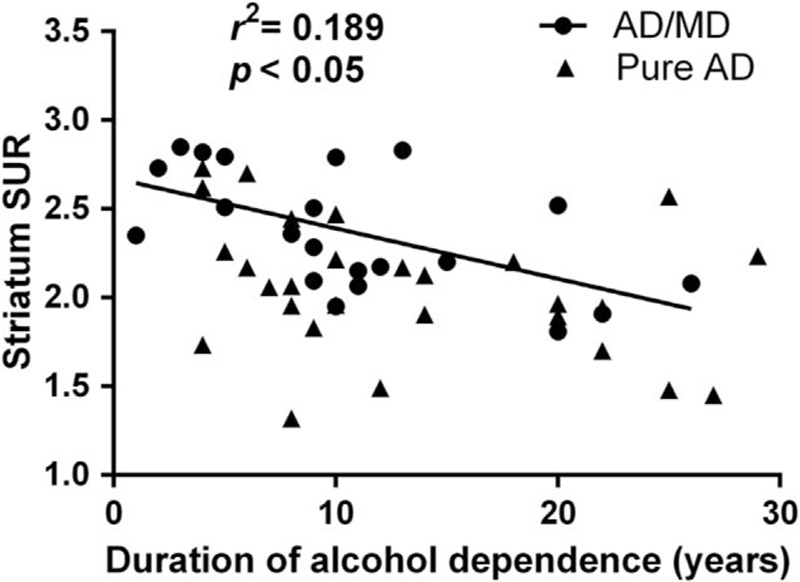
Graph showing correlation of striatal SUR of [^99m^Tc] TRODAT-1 with duration of alcohol dependence (years). Significant association between duration and striatum SUR existed in alcohol-dependent individuals. The coefficient of determination (*r*^2^) is 18.9% for the alcohol dependent group (n = 49) between duration and striatum SUR. SUR = specific uptake ratio.

When analyzing the relationship between striatal SUR, years of AD, and amount of daily alcohol intake, we found a significant association between striatal SUR and years of AD (rho = −0.455, *P* = 0.001) (Fig. 2), but no association between striatal SUR and amount of daily alcohol intake (rho = 0.157, *P* = 0.280).

**Figure 3:**
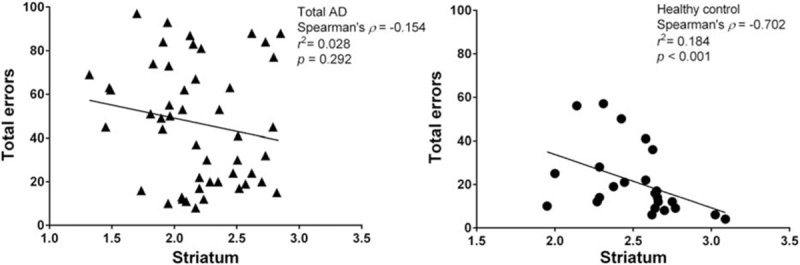
Graph showing correlation of striatal-specific uptake ratio of [^99m^Tc] TRODAT-1 with total errors. Significant correlation between these parameters existed in healthy controls, but not in AD group. AD = alcohol dependence.

The relationship between WCST tests and striatal DAT availability in patients with pure AD by Spearman correlation revealed that a marginal association was observed for total errors (rho = −0.407, *P* = 0.032), perseverative errors (rho = −0.385, *P* = 0.043), and categories completed (rho = 0.379, *P* = 0.047) (Table 4). Nevertheless, these marginal results were insignificant after Bonferroni correction (conservative *P* value would be 0.05/24 = 0.0021). In addition, there is no association between DAT availability and the WCST parameters in both total AD and AD/MD groups. The healthy subjects reached statistical significance between striatal DAT availability and total errors (rho = −0.702, *P* < 0.001) (Fig. 3), perseverative errors (rho = −0.688, *P* < 0.001), failure to maintain set (rho = −0.408, *P* < 0.001), and nonperseverative error (rho = −0.578, *P* = 0.003) (Table 4).

**Table 4:**
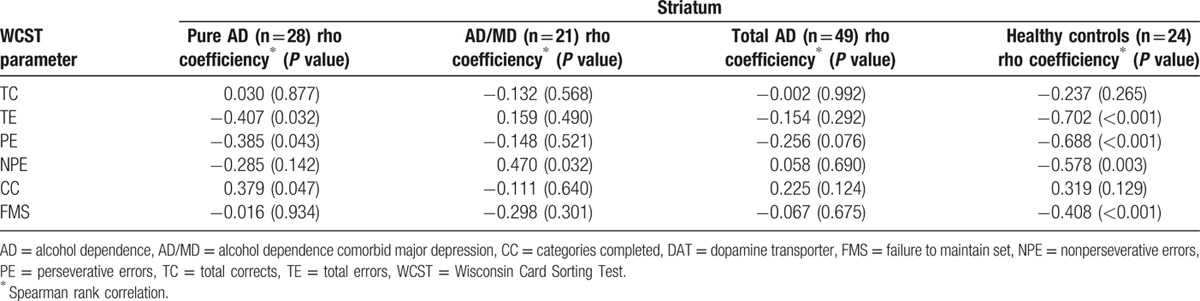
Association between WCST parameters and DAT availability in pure AD patients, AD/MD, and healthy controls.

**Figure 4:**
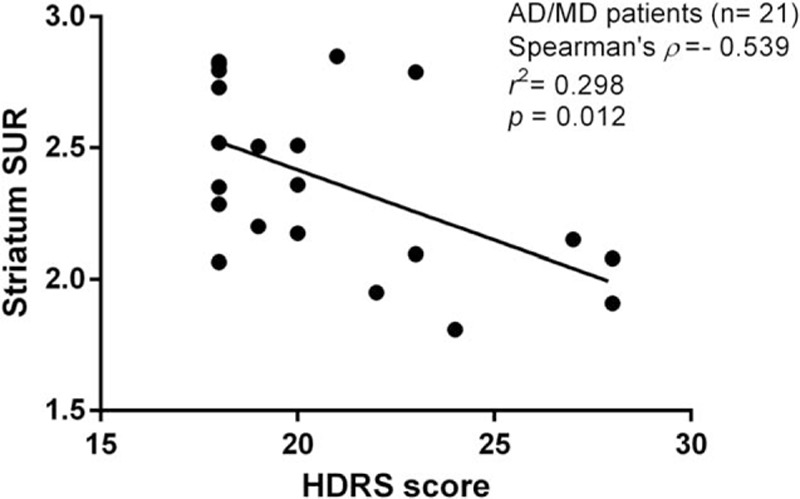
Graph showing correlation of striatal SUR of [^99m^Tc] TRODAT-1 with HDRS in AD/MD group. Significant association between HDRS score and striatum SUR existed in AD/MD individuals. The coefficient of determination (*r*^2^) is 29.8% for the AD/MD group (n = 21) between HDRS score and striatum SUR. AD/MD = alcohol dependence comorbid major depression, HDRS = 17-item Hamilton Depression Rating Scale, SUR = specific uptake ratio.

A significant negative correlation between the HDRS scores and DAT availability was found in the AD/MD group (rho = −0.539, *P* = 0.012 for the striatum; rho = −0.435, *P* = 0.048 for the putamen; and rho = −0.558, *P* = 0.009 for the caudate), but not in the pure AD and healthy control groups (*P* > 0.05) (Fig. 4).

## 4 Discussion

### 4.1 Brain DAT availability in patients with AD

DAT dysfunction is an important factor in the pathogenesis of both AD and MD; however, previous studies have not examined DAT availability in patients within different subgroups of AD. This study showed significantly lower DAT availability in the brains of male patients with AD compared with those of healthy controls. Of the different subgroups we examined, this study found that the pure AD group exhibited the lowest DAT availability compared to that in the AD/MD and control groups. Our results are similar to prior neuroimaging studies in patients with AD,^[8,10,11]^ but differ from those of others.^[26,27]^

These conflicting results may be attributed to several factors. First, the timing of performing brain imaging during different periods may influence the results of DAT availability in patients with AD. A human study using [^123^I] β-CIT SPECT on patients with AD found a decrease in DAT availability during the withdrawal period followed by a significant increase after a period of abstinence.^[9]^ The results of that study indicated that DAT availability might be transiently reduced in the alcohol withdrawal state and gradually increased after a period of abstinence. In order to exclude the time-effect on brain DAT availability, our patients were enrolled during continued drinking status and the brain imaging was performed within the early withdrawal state (within 72 h of the last drink). Thus, the time factor is unlikely to have interfered with our results. Second, the selection of the radioligand may influence the analysis of data. For example, [^123^I]-β-CIT is not a specific radioligand for DAT because it also has affinity for serotonin^[28]^ and norepinephrine.^[29]^ The specificity of the radioligands and semiquantification techniques will provide more objective information and diagnostic accuracy on DAT availability.^[30]^ Therefore, it is important to choose highly specific tracers in the investigation of DAT availability. At present, ^99m^Tc-TRODAT-1 is the most specific radioligand for DAT,^[31]^ and it seems to be a suitable SPECT radiotracer for further imaging studies of DAT availability in AD and MD. Third, patients with AD are usually comorbid with depressive disorder,^[20]^ and previous ^99m^Tc-TRODAT-1 SPECT studies suggest that DAT availability is higher in depressive subjects than in healthy controls.^[32,33]^ Hence, major depression (MD) is one of the confounding factors in the investigation of DAT availability in patients with AD, but previous brain DAT imaging studies could not exclude these confounding factors.^[10,11]^ In order to evaluate the influence of depressive effects on brain DAT activity, the present study recruited patients with AD/MD and pure AD. Thus, the results of incongruent reduction of DAT availability in different subgroups of AD (positive results in pure AD, and negative results in AD/MD, Table 2) may explain previous controversial results. Although this is the first study to examine brain DAT availability of patients with AD in different subgroups, the confounding factors of different personality and MD in patients with AD should be further investigated.

### 4.2 Age, sex, and smoking status on DAT availability

Age, sex, and smoking behavior may have confounding effects on DAT availability. Our results found a significant negative correlation between brain DAT availability and age in healthy controls (rho = −0.485, *P* = 0.016), which is similar with the findings of other reports.^[21,34]^ However, we did not find a strong relationship between striatal DAT availability and age in patients with AD/MD (rho = −0.325, *P* = 0.150). These data indicate that alcohol and depression may play a more significant role than age in influencing DAT availability. With respect to sex effects, prior studies have reported higher DAT availability in female.^[21,35]^ This study showed no age difference between patients with AD and healthy controls and only enrolled male subjects, thus abolishing age and sex as confounding factors in this study.

Cigarette smoking may have indirect effects on the mesolimbic dopaminergic system.^[36]^ Yang et al^[37]^ and Newberg et al^[38]^ reported a decrease in striatal DAT availability in smokers compared with nonsmokers; however, this association was not found in another study.^[39]^ The present study found no correlation between the number of cigarettes smoked per day and striatal DAT availability (rho = −0.269, *P* = 0.062, data not shown) in the total patients with AD. However, the effect of smoking on DAT availability remains controversial and needs to be investigated further.

### 4.3 Cognitive function and striatal dopamine dysfunction in patients with AD

The WCST is a neuropsychological assessment tool widely used to assess frontal lobe activity and corticostriatal pathway function.^[40]^ The DAT has been reported to be associated with cognitive functions in previous studies.^[41,42]^ Spatial working memory is impaired in DAT knockout mice.^[43]^ Selective damage of the dopaminergic neurons in rats can lead to cognitive deficits, particularly when the mesocorticolimbic pathway of the dopaminergic system is involved.^[44]^ This study found a negative correlation between brain DAT availability and the performance of WCST tasks in the control group (Fig. 3, Table 4). These results are consistent with our previous study^[10]^ and other studies in human,^[45,46]^ which suggest that striatal dopaminergic function may play an important role in performance on cognitive tests.

Low DAT availability and cognitive dysfunction were found among different AD subgroups, but we showed no relationship between brain DAT availability and performance on the WCST in patients with total AD and AD/MD. Although the early withdrawal symptoms may 1 possibility to influence the performance on WSCT tasks among patients with AD, other possibilities such as inflammatory factors and genetic heterogeneity need to further investigated in the future.

### 4.4 Association between the HDRS scores and DAT availability in patients with AD

This study found that the HDRS scores are negatively correlated with DAT availability in patients with AD/MD over the caudate, the putamen, and the striatum. These results fit the “low dopamine hypothesis” for explaining depressive symptoms and we suggest that the dopamine-depletion-induced down-regulation of the DAT occurs during depressive episodes in patients with AD/MD. The negative correlation is consistent with previous studies of depressive symptoms in Parkinson disease^[47,48]^; however, the relationship could not be found in patients with MD only^[49,50]^ or patients with AD only.^[51]^ Although prior studies analyzed the relationship between depressive symptoms and DAT availability in the brain, these studies used different patient groups, which might have affected the measures of dopamine availability in the brains of patients with depression. MD is a common comorbidity in patients with AD, but there are no studies on the relationship between depressive severity and DAT availability in patients with AD comorbid with a current major depressive episode. Our results suggest that decreased DAT availability (density) in the striatum may play an important and necessary role in the development of depressive symptoms in patients with AD/MD. Therefore, dopaminergic-related antidepressants may be beneficial in the treatment of depressive alcoholism.

## 5 Limitations

Result of the present study should be interpreted with caution because of the following limitations. First, the small sample size of this study reduced statistical power. In order to prevent possible type I errors, we recruited only male patients within 2 subgroups of AD from a Han Chinese population, thus limiting the effects of ethnicity and multifactorial diversity. Second, the study design used a cross-sectional method. Some of the patients initially diagnosed with MD might be more accurately diagnosed with bipolar disorder after a subsequent manic or hypomanic episode. Third, our subjects with AD had taken lorazepam to prevent withdrawal syndromes before SPECT imaging, and there is evidence to suggest that benzodiazepam may interfere with levels of extracellular dopamine.^[52]^ Fourth, patients with AD/MD may have differences in length of illness, duration of episode, quality of sleep, and severity of symptoms, which may have influenced SUR values.

## 6 Conclusions

The present findings suggest that the incongruent reduction of DAT availability in different subgroups of AD may play a pathophysiological role in the development of AD, and different drinking behaviors may influence the stabilization of brain dopamine levels. We found that decreased DAT availability may be associated with neurocognitive deficits, and that DAT availability may play an important role in normal neurocognitive function. Moreover, the availability of brain striatal dopamine may influence the severity of depressive symptoms in patients with AD/MD. To confirm this, further studies are required to establish consistency of the incongruent reduction of DAT availability in different clinical subgroups.
